# Neoantigens: new hope for cancer therapy

**DOI:** 10.3389/fonc.2025.1531592

**Published:** 2025-03-11

**Authors:** Yitong Hu, Tengda Zhou, Ping Cai, Zihao He

**Affiliations:** ^1^ Health Science Center, Ningbo University, Ningbo, Zhejiang, China; ^2^ Ningbo No.2 Hospital, Ningbo, Zhejiang, China

**Keywords:** neoantigen, tumor, immunotherapy, prediction of neoantigen, new treatment

## Abstract

As research into tumour immunotherapy continues to accelerate, new frontiers are being revealed in the field of cancer treatment. A significant focus has been drawn to neoantigen-based personalised tumour vaccines, a pioneering immunotherapy. This approach involves the use of genetic mutations that are unique to tumor cells to custom-design personalized tumor vaccines. These vaccines elicit an immune response that is specifically directed at targeting and eliminating cancer cells. The incorporation of neoantigens, arising from mutations within tumor cells, confers a distinct advantage to personalized tumor vaccines in terms of precision and the mitigation of adverse effects. However, the intricate pathways from antigen presentation to the activation of tumor immunogenicity remain to be elucidated. This paper primarily delves into the origins and characteristics of neoantigens, and also neoantigen prediction, highlights existing screening methods, and addresses the limitations of current approaches. It is hoped that this review will act as a catalyst, accelerating the understanding of relevant knowledge and illuminating research hotspots for scientists poised to venture into neoantigen research.

## Introduction

The treatment of malignant tumours is considered to be one of the most significant medical challenges of the 21st century. In recent years, the advent of innovative cancer therapies has illuminated potential pathways for surmounting the daunting challenge of tumours to human health. Beyond the conventional treatment modalities such as radiotherapy, chemotherapy, and targeted therapy, immunotherapies have emerged as a pivotal approach, garnering significant attention in research endeavours, with cancer vaccines emerging as a central focus in current research (1).

Nevertheless, numerous challenges persist in the realm of cancer vaccines, including the fact that the clinical efficacy of vaccines is only modest, the selection of optimal antigens is problematic, issues are encountered with vaccine delivery, and immune evasion tactics are employed. However, recent studies have demonstrated encouraging results with personalised neoantigen treatments in Phase I clinical trials, indicating a novel direction for the advancement of cancer vaccines (2).

The antigens contained within tumour vaccines can be categorised into four distinct classifications: Tumor-Associated Antigen (TAA), Tumor Specific Antigen (TSA), Cancer-Testis Antigen (CTA), and Viral Antigen. Traditional Tumor-Associated Antigens are characterised by their expression in both normal somatic cells and tumor cells. In contrast, tumor-specific antigens, also known as neo-antigens, are not expressed in somatic cells but are specifically mutated and generated in tumor cells. They feature low side effects and are not restricted by central tolerance in the thymus. Cancer-testis antigens, on the other hand, are primarily expressed in testes and embryonic tissues, with low levels observed in other adult tissues. However, these antigens can be reactivated and expressed in various tumors. Finally, viral antigens are a consequence of virus-infected tumours and are expressed in tumour cells, thus serving as vaccine targets.

The clinical successes of neoantigens are not a matter of chance, as their effectiveness and the mechanisms underpinning them are being steadily corroborated (3). However, the development of neoantigens is encumbered by a number of challenges, including the necessity for complicated preparation, the imposition of high costs, and the involvement of time-consuming processes, in addition to the occurrence of high false-positive rates in screening. However, the advent of big data and artificial intelligence has engendered a favourable environment for the development of neoantigen screening tools. The utilisation of substantial machine learning data and optimised algorithms holds the potential to provide valuable guidance for neoantigen screening.

This article provides a comprehensive review of the extant literature on neoantigens, with a particular focus on recent research developments. The aim of this review is to facilitate the initiation of scientific research in this field by potential contributors.

## The mechanism of T cells and neoantigen

T cells represent a pivotal component of the human immune system, endowed with the capacity to discern non-self peptides that are presented on the cell surface via the Major Histocompatibility Complex (MHC). In humans, MHC molecules are also known as Human Leucocyte Antigens (HLA). T cell recognition represents the most selective stage in the process of antigen presentation. T cells initially develop from pluripotent hematopoietic stem cells in the bone marrow, maturing in the thymic microenvironment. Prior to migrating from the thymus to the peripheral blood and immune organs, T cells undergo positive and negative selection, both of which are crucial for their development. Positive selection confers MHC restriction, while negative selection ensures self-tolerance. Only T cells that successfully pass both selections develop into mature T cells; otherwise, they undergo apoptosis (4).

**Figure 1:**
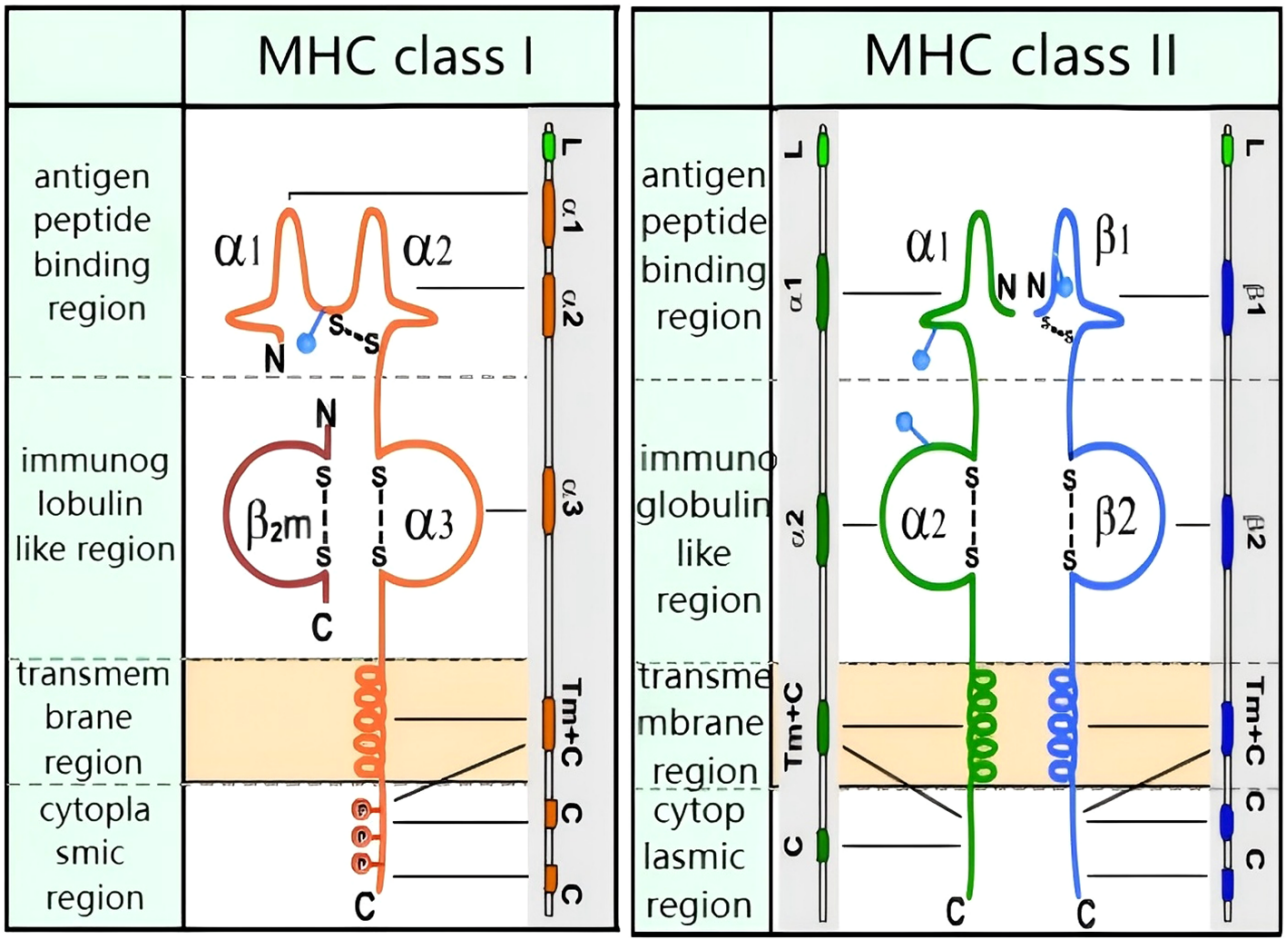
Molecular structure of MHC class I and MHC class II: MHC class I: transmembrane heavy chain (orange) non-covalently bound to β2-microglobulin (red). MHC class II: transmembrane α-chain (green) and β-chain (blue) connected by disulfide bonds. Both classes feature antigen-binding grooves (α1/β1 for II, α1/α2 for I).

The MHC is critical to the process of antigen presentation and is a key component of the immune response system. MHC subtypes are numerous and polymorphic, and are generally classified into MHC Class I and MHC Class II molecules (see **Figure 1**). As demonstrated in **Figure 1**, both have four functional regions: the peptide-binding region, the immunoglobulin-like domain, the transmembrane region, and the cytoplasmic region. MHC Class I molecules consist of a heavy chain (alpha chain) and a light chain (beta chain), while MHC Class II molecules comprise an alpha chain and a beta chain.

MHC Class I molecules primarily engage with endogenous antigenic peptides, thereby establishing the foundation for their subsequent recognition upon binding to CD8 T cells. In contrast, MHC Class II molecules chiefly interact with exogenous antigenic peptides, with these complexes being identified by CD4 T cells upon binding. However, tumor cells have been observed to employ a deceptive tactic, suppressing the expression of MHC on their surface, thus undermining the presentation of antigens to CD8 T cells. This results in a state of immunosuppression, where CD8 T cells are rendered incapable of eliminating tumor cells.

**Figure 2:**
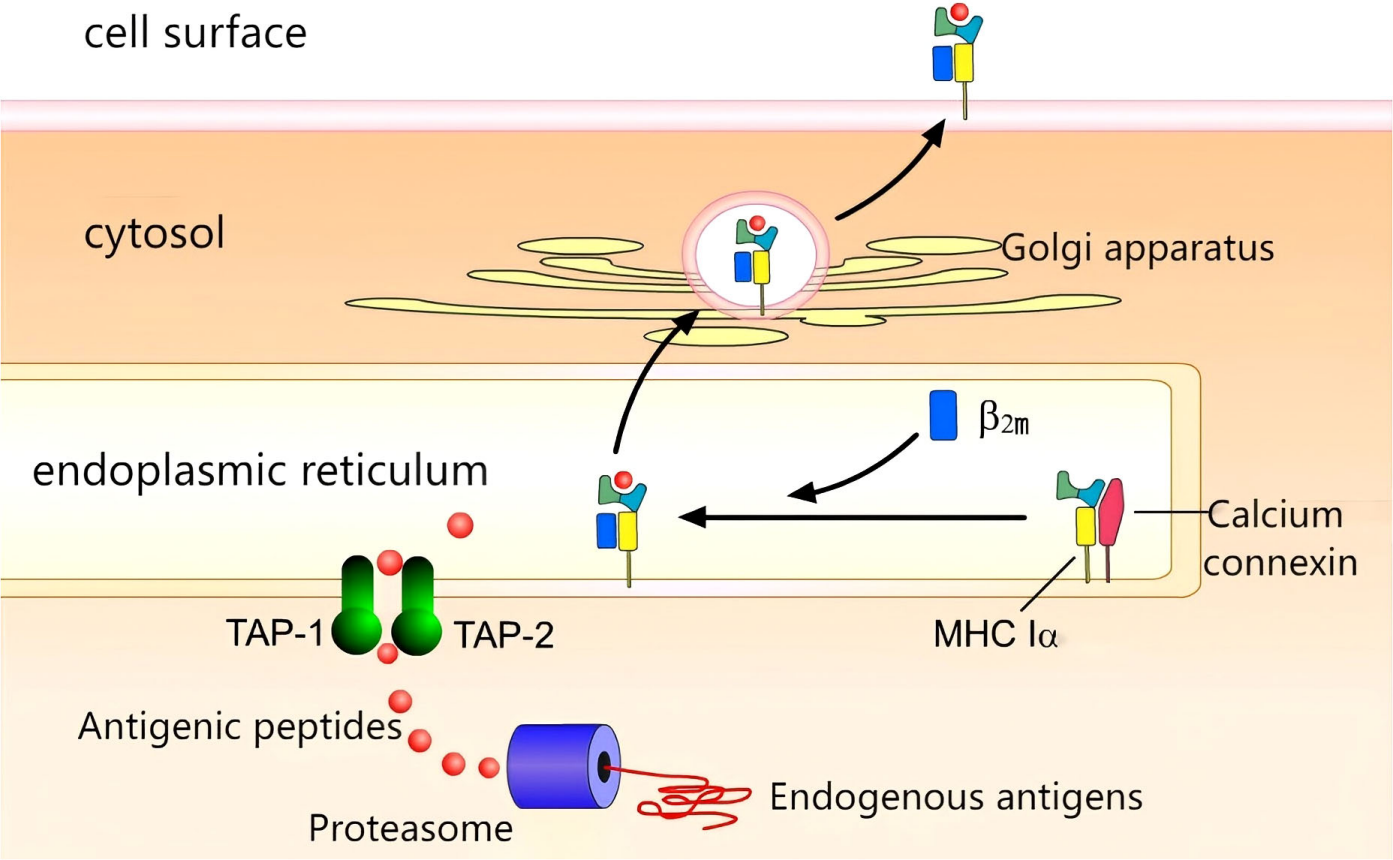
Antigen presentation:Proteasomal degradation generates peptides transported via TAP-1/2 to ER. MHC I-peptide complexes assemble and traffic through Golgi to cell surface.

The complex process of antigen presentation is illustrated in **Figure 2**. Within the context of tumour cells, aberrant proteins undergo degradation into peptides by the action of the proteasome. Concurrently, MHC Class I molecules, with the assistance of calnexin, are synthesised. The peptides then embark on a journey through the endoplasmic reticulum via the transporter associated with antigen processing (TAP), culminating in the formation of an antigen peptide-MHC Class I complex. This complex then traverses the Golgi apparatus, reaching its final destination on the cell surface, where it binds to the T-cell receptor (TCR) (5).

The binding of the antigen to the MHC complex on the surface of the antigen-presenting cell (APC) initiates a series of reactions that result in the transformation of CD8 T cells into active cytotoxic T lymphocytes (CTLs). The body thus initiates an immune response through this cascade of reactions. Equipped and ready, CTLs can then unleash their cytotoxic activity, targeting and eliminating tumor cells (6).

## Tumour neoantigens

Contemporary scientific research has elucidated that the genesis of tumours originates from mutations in the genes of somatic cells, which accumulate over time. Genes impacting tumorigenesis are divided into two categories: oncogenes and tumor suppressor genes. In the context of normal cellular processes, these genes play a pivotal role in orchestrating cell proliferation. However, in the event of these genes undergoing aberrant mutations, the equilibrium that governs cell proliferation regulation becomes compromised, thereby paving the way for the emergence of tumors (7).

With the advent of epigenetics, it has been unveiled that the factors underpinning carcinogenesis are multifaceted, the processes incredibly complex, encompassing not just alterations in genetic material but also abnormal epigenetic modifications of histones or DNA (8), capable of triggering the transformation of normal cells into cancerous ones.

The hallmark of neoantigens is their exclusive expression in tumour cells. These short peptides, which are the product of the degradation of an abnormal protein resulting from somatic mutations, are distinctive features of neoplastic cells (9).

**Figure 3:**
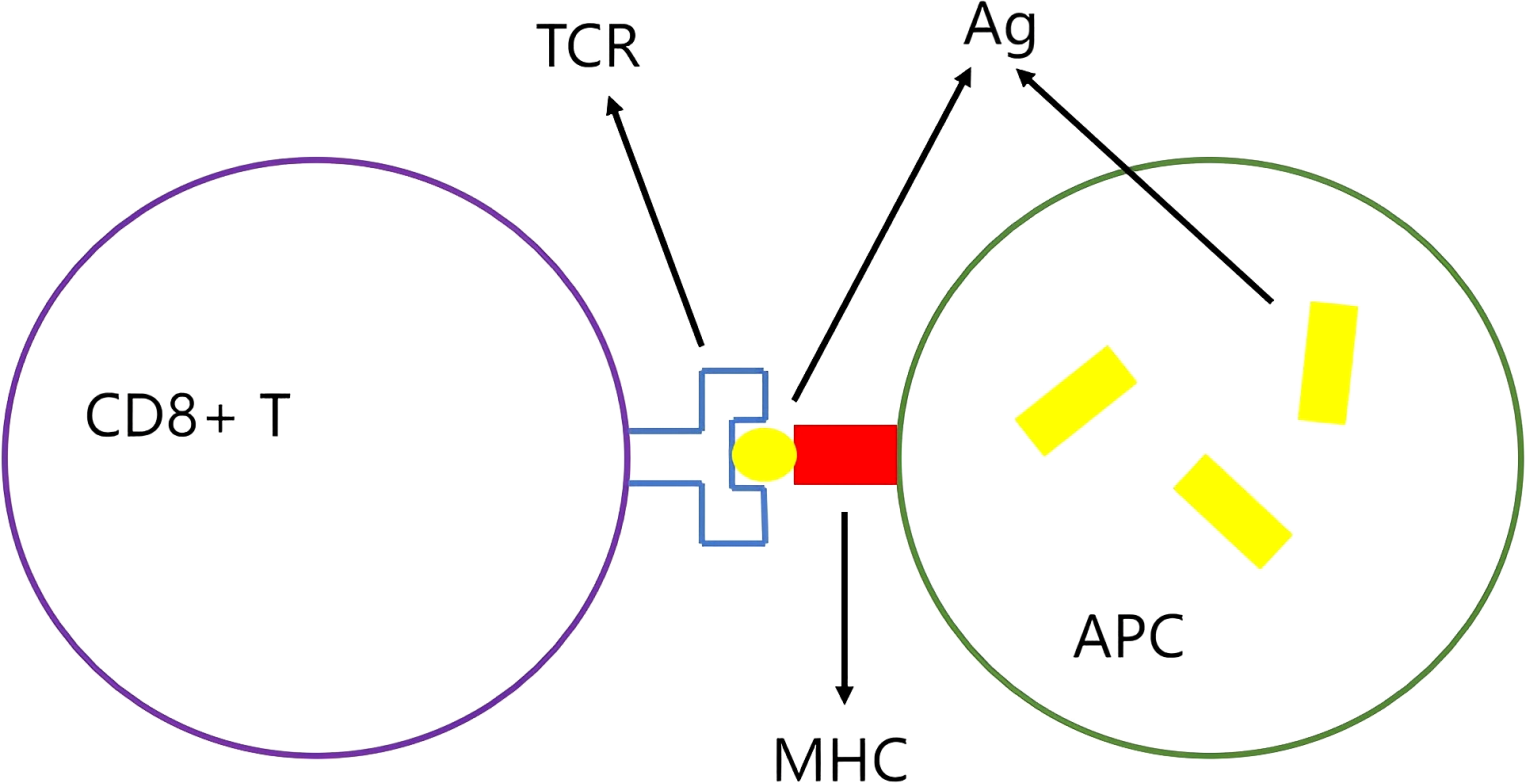
The antigen-MHC complex is recognized by the TCR: The antigen (Ag) is presented by the antigen-presenting cell (APC) to the T cell receptor (TCR) via the major histocompatibility complex (MHC), which activates CD8+ T cells.

The process by which antigen engages with the T-cell receptor (TCR) is delineated in **Figure 3**. These short peptides are identified by antigen-presenting cells (APCs). Short peptides that adhere to the major histocompatibility complex (MHC) binding motif are pinpointed by the TCR, thereby initiating an antitumor immune response. Armed with this recognition, T cells can then wield their power to lyse tumor cells, unleashing additional tumor neoantigens. This cascade of events can ultimately culminate in a broadened antitumor immune response (10).

It is imperative to acknowledge that the process by which neoantigens galvanise tumour immunity is influenced by a multitude of factors. Research elucidates that the crosstalk between tumours and the immune system shapes the immunogenicity of neoantigens. The dynamic interplay between the tumor and the immune system can be broadly categorised into three phases:First Phase (Elimination Phase): Under the leadership of the immune system, antigens are targeted and subsequently eliminated, particularly those with high antigenicity, such as tumor cells;Second Phase (Equilibrium Phase): Tumor cells with less conspicuous antigenicity evade the immune system’s onslaught. Due to the influence of the adaptive immune system, they retreat into a dormant state. The dynamic equilibrium between the production and clearance of tumor cells is a hallmark of this phase. In this phase, the immune system selects and enriches tumor cells that can thwart the immune system’s killing, a phase also designated as immune editing. Finally, the Third Phase (Escape Phase) In this phase, refined and reconfigured tumor cells evade the immune system’s recognition, forging an immunosuppressive tumor microenvironment. The balance is thus disrupted, and the tumor cells initiate a rapid proliferation (11).

## Progress on research into neoantigens

In 2014, Ton N. Schumacher et al. published a study on an advanced cholangiocarcinoma patient whose symptoms showed significant improvement following treatment with a neoantigen vaccine. This study demonstrated the research value of neoantigens in tumour treatment (12).

In 2017, Ugur Sahin et al. published two articles with a close relation to neo-antigens. The first of these articles involved the use of neoantigen-personalised peptide vaccines in the context of melanoma research. The results demonstrated that patients receiving neoantigen vaccine treatment exhibited phenomena such as reduced recurrence and significant tumour shrinkage (13).

The second research detailed the administration of personalised peptide vaccines based on neoantigens to six melanoma patients. Notably, four of these patients exhibited no recurrence 25 months following vaccine inoculation (2).

In 2018, Ugur Sahin and others, who wield significant influence within the domain of tumour research, undertook a thorough and exhaustive review of recent research on neoantigens. Through meticulous analysis of a substantial amount of research data, clinical cases and cutting-edge achievements, they provided a positive evaluation of the exciting effects shown by neoantigen-based tumour treatments. This has engendered a renewed sense of optimism among patients, who had previously been constrained by the limitations of conventional tumour treatments (14).

## Prediction and screening of neoantigen

The advent of gene sequencing technology has led to significant advancements in the field, with next-generation sequencing (NGS) now capable of performing whole exome sequencing (WES) with unparalleled precision. Nevertheless, a critical limitation persists: single-base-pair nucleotide mutations frequently go unidentified, which significantly complicates reliance on WES for the reliable prediction of tumour neoantigens (15).

Whilst the WES does not offer a direct solution to the issue of neoantigen screening, its development has created additional pathways for neoantigen identification, thereby rendering computer-aided neoantigen prediction a possibility. The methods for neoantigen prediction and screening can generally be categorized into four types.The initial method employs a comprehensive strategy involving MHC and antigen affinity screening. Initially, WES sequencing is performed on both normal and tumour cells from the patient to uncover non-synonymous single nucleotide variants (nsSNVs), insertion/deletion sites, and fusion genes (16), precisely pinpointing mutation sites. However, it should be noted that due to the high mutational load of tumour cells, over 50% of mutations are not further transcribed, thus rendering WES sequencing ineffective in addressing this issue. Consequently, RNA-seq on the initially identified mutation sites is essential to confirm transcription (17). Subsequent analysis of the patient’s HLA typing through WES and RNA-seq on peripheral blood cells employs prediction tools to assess the affinity of HLA and antigens. The top-scoring antigens are then subjected to experimental validation to identify candidate neoantigens. The merits of this method are its expediency and capacity for large-scale parallel prediction; however, it is accompanied by the necessity for substantial data support, exhibits low prediction accuracy, and necessitates downstream experimental validation.

The second method is centred upon the screening of neoantigens through the utilisation of short gene tandem sequences. The process and steps for obtaining nsSNVs and MHC typing are similar to method one, but instead of predicting MHC-antigen affinity with prediction tools, several genes containing mutation sites are concatenated to create short tandem minigenes (TMGs). These TMG control the expression of mutated genes into peptides of around nine amino acids in length. These TMGs are then spliced onto plasmids for *in vitro* transcription and expression. Finally, the immunogenicity of these mutated genes expressed as peptides is verified through T-cell reactivity analysis (18).

The third method utilises neoantigen screening from databases. In addition to gene sequencing methods, there are numerous existing literature and databases that have introduced antigen epitopes proven to have immunogenicity. The method involves the identification of high-frequency mutation sites from existing resources with a view to ultimately uncovering potential neoantigen epitopes (19). The primary benefit of these antigens is that they have already undergone experimental validation. However, the disadvantage is that the coverage is extremely limited, catering to a small number of patients.

Method four employs the process of predicting neoantigens through the execution of mass spectrometry elution experiments. Specifically, an elution experiment is performed on antigen peptides bound to MHC molecules in tumor tissues. The immunogenicity of the antigens is then analysed based on the resulting mass spectrometry data.

The core experimental workflow is outlined as follows. Initially, peptides bound to MHC molecules are to be immunopurified. Subsequent to this, high-resolution tandem mass spectrometry is to be performed under liquid-phase conditions. Subsequent to this, a comprehensive protein database is to be constructed. Subsequent to this, advanced protein quantification and identification analysis tools, such as Peaks (20) and MaxQuant (21), are to be utilised in conjunction with the constructed database to thoroughly analyse the mass spectrometry data and pinpoint mutated peptides. Finally, the immunogenicity of the peptides must be validated through *in vitro* assays.As this method encompasses a series of information from antigen processing to presentation, it avoids false positive predictions stemming from these intermediate processes, thus significantly enhancing the accuracy of neoantigen prediction (22). The merits of this approach are evident in its ability to directly detect the final neoantigen with high precision. However, it is important to note the limitations imposed by mass spectrometry, which results in a higher rate of false negatives.

## Problems and solutions of neoantigen

The preceding section provides a detailed exposition of the four predominant methodologies for neoantigen screening. The advent of bioinformatics and the subsequent arrival of the big data era have engendered a proliferation of data, thereby creating a wealth of opportunities for machine learning in artificial intelligence algorithms and models that predict MHC molecule-antigen affinity. Consequently, the focus of neoantigen screening has transitioned to method one: screening through MHC and antigen affinity. It is acknowledged that numerous tools are currently available for predicting MHC-antigen affinity.

NetMHCpan-4.0 is a notable example of an accurate neoantigen prediction tool. The author hypothesises that the majority of contemporary prediction tools are constructed using binding affinity (BA) data to formulate machine learning models. However, BA is only capable of representing a single event, namely whether the MHC and peptide bind tightly. While this event constitutes the most selective step in the entire antigen presentation process, BA does not reflect the true immunogenicity of an antigen. Prediction tools that are modeled using BA data frequently exhibit a high false positive rate, and the underlying reason for this is not difficult to explain. The antigen presentation process encompasses numerous other steps, including peptide processing, TAP transport, and ERAP trimming, which are not adequately captured by BA data. Mass spectrometry (MS) data can incorporate this information, but unfortunately, the amount of MS data in the Immune Epitope Database (IEDB) (23) vastly outmatches that of BA data. Consequently, the author amalgamated MS and BA data to establish a neoantigen prediction model employing an Artificial Neural Network (ANN) method. The model comprises four input categories based on peptide length: L ≤ 8, L=9, L=10, and L≥11, and two outputs: BA prediction and MS prediction. The model can also predict the optimal peptide length for antigens binding to different MHC types based on MHC typing. However, given the substantial disparities in affinity scores between different MHC subtypes and antigens, establishing a unified evaluation benchmark presents a challenge. The author converted the scores into a percentile Rank, and the node with the optimal specificity and sensitivity based on the Rank score is utilised to screen for neoantigens. Peptides with a Rank value in the topmost 0.5% are deemed to have robust binding ability to MHC molecules; peptides with a Rank value between 0.5% and 2% exhibit weak binding ability; and peptides with a Rank value exceeding 2% are incapable of binding to MHC molecules (24). Despite the integration of BA and MS data in the NetMHCpan-4.0 training set, the scarcity of MS data gives rise to the problem of false positives in prediction outcomes.

In a recent publication in Cell, Daniel K. Wells et al. introduced the use of multiple tools to predict the immunogenicity of tumour epitopes and analysed some key parameters that can improve the accuracy. The author coordinated 25 research teams to predict neoantigens for the same cancer patients; subsequently, 608 peptides were selected from all the results for experimental testing, among which 37 were identified as immunogenic. Statistical outcomes revealed that each team correctly predicted 1-20 immunogenic peptides among their top 100 peptides, but the ranking of these immunogenic peptides varied significantly among teams.

Subsequent to this, the 37 immunogenic results were subjected to rigorous scrutiny from multiple dimensions, encompassing the duration and strength of binding to MHC molecules, peptide expression abundance, hydrophobicity, and mutation site locations. The analysis revealed that immunogenic results exhibited enhanced binding affinity, elevated expression levels, increased binding stability, and diminished hydrophobicity. Furthermore, it was observed that mutation sites in peptides frequently occurred at the third amino acid, while the second amino acid was rarely mutated. The incorporation of this information into prediction tools could lead to the exclusion of 93% of non-immunogenic peptides (25).

The critical parameters outlined in this study can be employed as new features in future machine learning models, with the potential to generate highly accurate prediction models that exhibit a reduction in false positives. It is acknowledged that there are other tools for predicting MHC-peptide affinity, including PSSMHCpan (26), NetMHCpan (27),antigen.garnish (28) and BOTA (29), amongst others.

The aforementioned prediction tools predominantly concentrate on neoantigen prediction stemming from somatic mutations that engender novel epitope peptides, a prevailing approach in contemporary neoantigen prediction research. However, such predictions frequently yield false positives. However, it is important to note that neoantigens can also arise from alternative pathways, such as the transcription of silenced genes in adult tissues, which have the potential to transform into tumour neoepitopes, and anomalous splicing of introns, which can serve as an additional source of tumour neoepitopes. These retained introns are frequently overlooked due to false negatives.

Van Allen’s research ensemble developed an algorithm that predicts retained introns predicated on RNA sequencing, capable of pinpointing translated intron segments situated between exons and terminators. The algorithm then forecasts the affinity of the corresponding intron peptide sequences to MHC molecules. The experimental corroboration of peptides with elevated predicted affinities has the potential to unveil novel candidate neoantigens. This methodology permits investigators to pinpoint neoantigens from a more expansive gamut of origins, contributing to a more nuanced comprehension of tumour immune responses. However, a limitation is its substantial dependence on the precision of RNA sequencing (30).

It has been hypothesised by certain scholars that, irrespective of the method employed, whether that be the comparison of differential genes between tumour and normal tissues through DNA sequencing or the screening for retained introns via RNA sequencing, neither of these methods provides a direct reflection of the binding strength of peptides to MHC molecules. Proteomics can provide a more direct indication of the strength of peptide-MHC binding. Consequently, the authors have devised a high-precision deep learning algorithm, AutoRT, geared toward underpinning neoantigen prioritization based on proteomics, enabling more sensitive and direct neoantigen prediction (31).

At present, the data sources employed for modelling neoantigen prediction models are predominantly experimental data. The acquisition of both BA data and MS data is time-consuming and resource-intensive, which undoubtedly delays the progress of neoantigen prediction research. Molecular docking is a technology that emerges from the intersection of biology, computer science, biochemistry and other disciplines. Its fundamental principle is the ‘lock and key’ model, which allows for the prediction of the strength of ligand-receptor interactions and binding conformations. While molecular docking scores may not perfectly align with BA data, the ranking ability of docking scores regarding binding tightness can be highly correlated with BA data (32). If the ranking information of peptides is predicted using molecular docking, this would expand the data for building neoantigen prediction models, which is of significant importance for neoantigen research.

**Figure 4:**
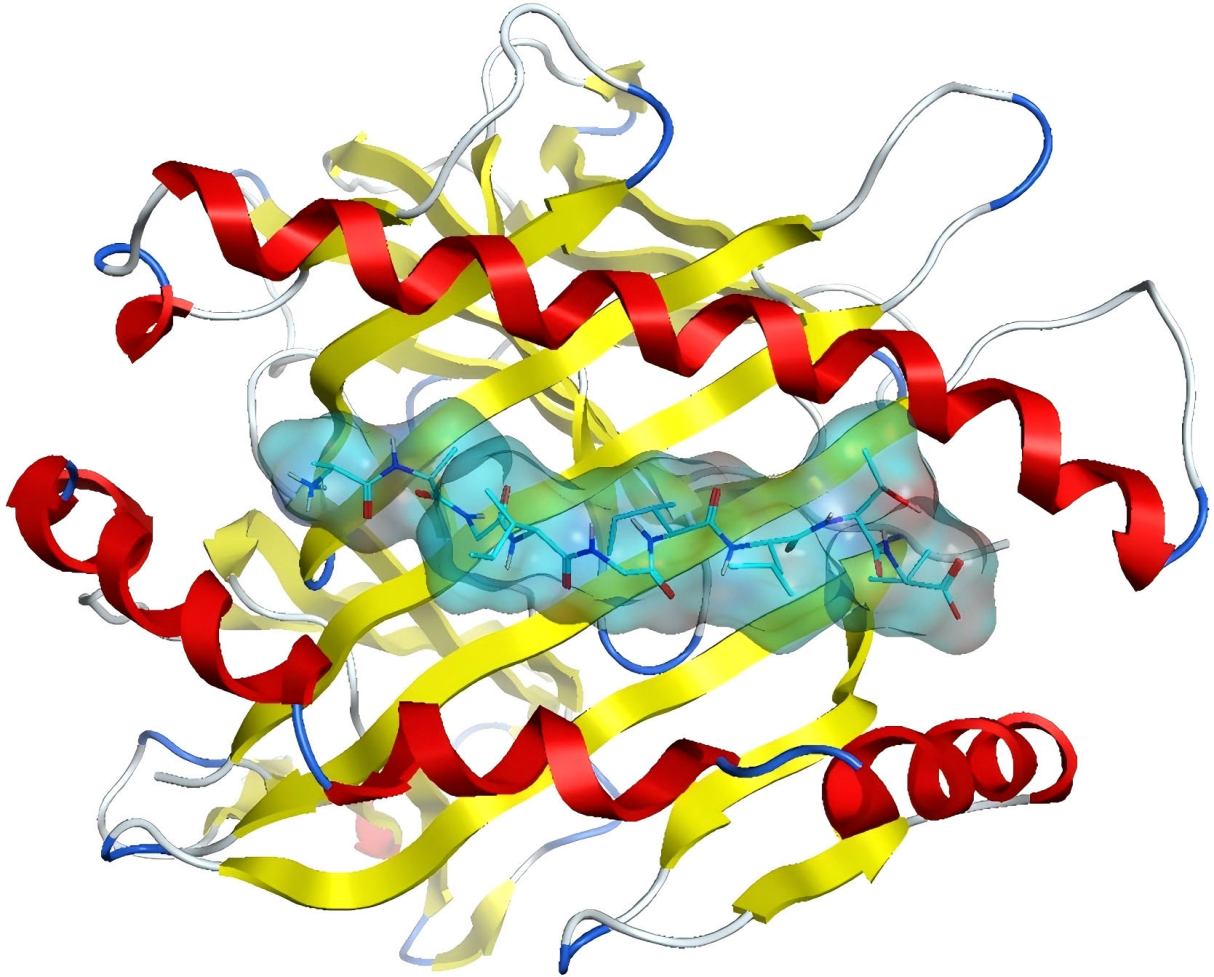
MHC binding pattern to nine peptides:Ribbon diagram depicts α-helices (red), β-sheets (yellow), and loops (white). Binding pocket surface with nine bound peptides (stick models).

Nevertheless, studies predicting neoantigens through molecular docking remain scarce. The authors have initiated preliminary investigations utilising the MOE molecular docking software (33). The crystal structure of a class I MHC molecule in complex with a nonamer peptide, which has been validated for strong binding affinity (PDB code: 3QFD), was selected for analysis. The retrieved crystal structure underwent a series of preprocessing steps, encompassing removal of water molecules, addition of hydrogens, elimination of heteroatoms, and energy optimization. The binding mode of the nonamer peptide to the MHC molecule is illustrated in **Figure 4**. The MHC molecule is depicted as a ribbon, with red signifying alpha-helices, yellow denoting beta-sheets, and white symbolizing loop structures. The nonamer peptide is illustrated using a stick model, while the binding pockets are rendered as surfaces.

**Figure 5:**
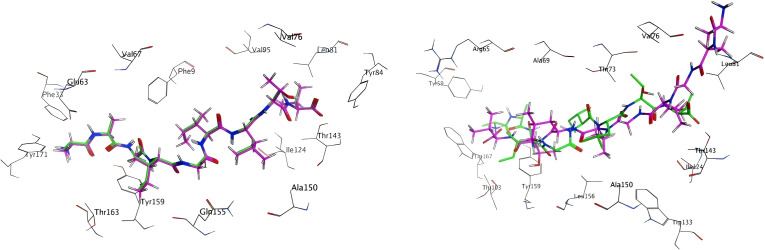
Docking results of the original ligand and the modeling ligand:Crystal structure peptides (green sticks) vs. MOE-docked conformations (purple sticks) demonstrating structural overlap (RMSD <2Å).

In order to ascertain the reliability of the docking software, a standard practice is to initially dock the original ligand prior to formal docking, in order to ascertain whether it can accurately replicate the crystal structure. The docking mode that was selected was protein-protein docking, which generated 20 conformations. The docking outcome conformation of the original ligand is depicted on the left in **Figure 5**. The green stick model signifies the conformation of the nonamer as per the crystal structure, and purple delineates the conformation of the docked structure. It is noteworthy that the two conformations can be superimposed virtually, thereby indicating that MOE’s protein-protein docking can adeptly reproduce the crystal structure.

A 3D model of a nonamer (AAGIGILTV) was constructed, with the ligand sequence mirroring that of the crystal structure. This was achieved using the Protein Builder module, followed by energy minimization. Subsequently, docking was executed using the identical docking mode, with the results illustrated on the right in **Figure 5**. It is evident from the figure that the docking outcome (green) does not align well with the crystal structure (purple), and an opposing orientation is even observed.

The top five docking score outcomes for both the original ligand and the modeled ligand are displayed in **Table 1**. It is evident that a lower score is indicative of a more robust binding between the ligand and the receptor. A clear distinction emerges between the original ligand and the modeled ligand, with the former demonstrating a significantly higher score.

**Table 1:** Docking scores of the original ligand and the modeling ligand.

Top 5	PROLIGAND score	Modeling ligand scores
**1**	-96.004 kcal mol^-1^	-38.142 kcal mol^-1^
**2**	-95.163 kcal mol^-1^	-29.396 kcal mol^-1^
**3**	-36.579 kcal mol^-1^	-28.466 kcal mol^-1^
**4**	-35.339 kcal mol^-1^	-28.255 kcal mol^-1^
**5**	-34.805 kcal mol^-1^	-27.757 kcal mol^-1^

**Figure 6:**
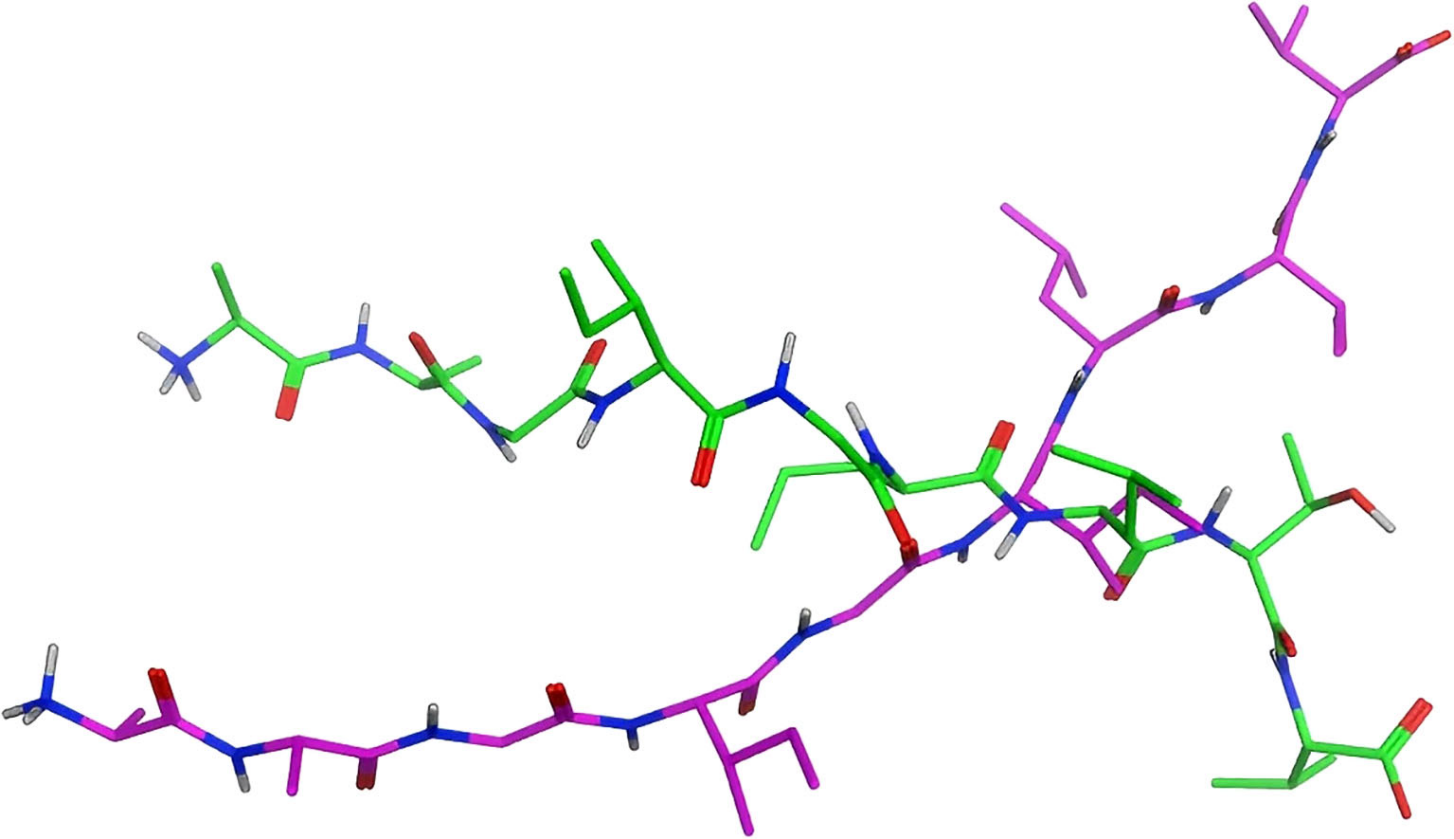
Conformations of the original ligand and the modeled ligand:Overlay of crystallographic ligand (green) and computational model (purple) configurations.

**Figure 7:**
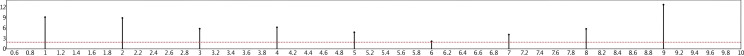
RMSD values of the original ligand and the modeled ligand:Cα RMSD between original and modeled ligands (mean 7.37Å). Dashed line at 2Å indicates conformational similarity threshold.

The configuration of the ligand is a pivotal factor in determining the outcomes of docking. As illustrated at the top of **Figure 6**, the green stick model exemplifies the conformation of the original ligand, whereas purple delineates the conformation of the modeled ligand. The root mean square deviation (RMSD) values for each Cα atom of both are delineated at the bottom of **Figures 6**, **7**, with an average RMSD value of 7.37Å. The red dashed line denotes an RMSD value of 2Å. Conformations can be regarded as comparable if the RMSD is below 2Å (34).This disclosure indicates that the peptide 3D structure engineered via the Protein Builder module and energy minimization diverges markedly from the crystal structure, possibly accounting for the substantial discrepancy in the docking conformations and docking scores.

The paucity of resolved MHC and peptide crystal structures underscores the utility of molecular docking as a potent methodology for forecasting docking conformations and binding affinity. As demonstrated by the aforementioned outcomes, it is evident that MOE’s protein-protein docking can replicate the crystal structure conformation and deliver commendable scoring, albeit with the precondition of possessing a peptide conformation that closely mirrors the authentic conformation. This is currently unattainable through the Protein Builder module and energy minimization alone.

The authors also attempted to obtain precise peptide structures via the utilisation of homology modelling utilities. However, the majority of modelling tools impose restrictions on the number of amino acids, with SWISS-MODEL requiring over 30 amino acids (35) and Robetta demanding over 26 amino acids (36), whereas the length of antigens typically ranges around 10 amino acids, rendering these modelling tools ineffective. Conformational searches were also executed by the authors, yet the outcomes remained disappointing.

The recent unveiling of AlphaFold by the DeepMind team has made it possible to predict the 3D structures of proteins with the same level of accuracy as that achieved by crystallographic techniques. It is anticipated that a similar tool for forecasting peptide 3D structures will be developed in the near future. At that juncture, molecular docking will undoubtedly assume a pivotal role in research concerned with predicting neoantigens.

## Future work

Genetic aberrations have been identified as a key factor in the development of tumours. However, these aberrant genes are also subject to surveillance by the immune system, contributing to the immunogenicity of the tumours. The advent of high-throughput sequencing technology has empowered researchers to expeditiously sequence patients’ DNA and RNA, thereby acquiring mutated gene loci. The employment of computational tools to predict the binding capacity of MHC and antigens facilitates the identification of potential neoantigens. Despite the plethora of prediction tools currently available, they commonly grapple with high false positive rates.

In the domain of tumour immunotherapy, the stimulation of cytotoxic T cells is of paramount importance. In this context, the process of antigen presentation constitutes the most discriminative phase, exhibiting a close interconnection with bioinformatics and computer science. Consequently, the development of neoantigen prediction methodologies with high sensitivity and specificity, capable of swiftly, cost-effectively and efficiently identifying neoantigen immunogenicity, is pivotal for advancing the application and development of tumour neoantigen therapy.

The advent of neoantigen-tailored immunotherapy promises to herald a new era in the fight against malignant tumours, with the potential to transform the landscape of cancer treatment.
